# Emotionally Intelligent People Use More High-Engagement and Less Low-Engagement Processes to Regulate Others’ Emotions

**DOI:** 10.3390/jintelligence10040076

**Published:** 2022-09-29

**Authors:** Hester Xiao, Kit Spencer Double, Sarah Ann Walker, Hannah Kunst, Carolyn MacCann

**Affiliations:** 1 School of Psychology, The University of Sydney, Sydney, NSW 2006, Australia; 2 Work and Organisational Studies, The University of Sydney Business School, Sydney, NSW 2006, Australia

**Keywords:** emotional intelligence, emotion regulation, reappraisal, expressive suppression

## Abstract

Existing research has linked emotional intelligence (EI) with intrinsic emotion regulation (processes people use to regulate their own emotions). However, there has not yet been an empirical examination of whether EI abilities relate to extrinsic emotion regulation (processes people use to regulate other people’s emotions). This study (N = 178 undergraduates) examines whether ability EI (as measured by the Mayer–Salovey–Caruso Emotional Intelligence Test) correlates with eight extrinsic regulation processes (as measured by the Regulation of Others’ Emotions Scale, including downward comparison, expressive suppression, humour, distraction, direct action, reappraisal, receptive listening and valuing). Total ability EI score is significantly positively correlated with three high-engagement processes (r = .24, .40, and .16 for reappraisal, receptive listening, and valuing) and negatively correlated with two low-engagement processes (r = −.30 and −.38 for downward comparison and expressive suppression). When all four EI branches predicted each regulation process in multiple regression, only emotion management significantly predicted downward comparison, receptive listening and valuing, and only emotion management and understanding predicted expressive suppression (no significant regression coefficients for reappraisal). We conclude that the drivers of EI/extrinsic regulation associations are engagement with the target’s emotion and the emotion management branch of EI.

## 1. Introduction

Emotion regulation is typically described as the processes people use to influence the type, intensity or timing of an emotion (Gross 1999), whereas emotional intelligence (EI) is the ability to perceive, use, understand, and manage emotions (Mayer et al. 2016). That is, emotion regulation is a set of behaviours (things people think or do) whereas EI is a set of personal characteristics (capacities people possess). Research shows that EI relates to which emotion regulation processes people use (e.g., emotionally intelligent people use less rumination and more positive reappraisal; Peña-Sarrionandia et al. 2015). However, research on EI/emotion regulation associations to date has focused solely on *intrinsic emotion regulation* (the processes we use to regulate our own emotions). Recently, there has been growing interest in *extrinsic emotion regulation* (the processes we use to regulate other peoples’ emotions). Several researchers have begun to characterize the processes involved in extrinsic emotion regulation, the predictors of extrinsic emotion regulation, and how extrinsic and intrinsic emotion regulation processes differ (Niven et al. 2011; Nozaki and Mikolajczak 2020). Our research adds to this growing area by examining for the first time whether EI abilities relate to *extrinsic* as well as intrinsic emotion regulation processes. Our major research question is whether the four ability branches of EI (emotion perception, facilitation, understanding, and management; Mayer et al. 2016) relate to eight extrinsic emotion regulation processes (MacCann 2022).

### 1.1. Extrinsic Emotion Regulation

The most influential model of emotion regulation is the temporal-process model, which describes emotions (and attempts to regulate them) as a process that unfolds over time (Gross 1999). Emotions occur in a situation–attention–appraisal–response sequence and can be regulated at different points along this sequence. Emotion regulation processes are classified in terms of five emotion regulation stages: (1) situation selection (changing the trajectory of emotion by approaching or avoiding particular situations); (2) situation modification (changing the situation to remove/reduce the emotional trigger); (3) attention deployment (changing the focus of attention towards or away from particular elements to change the emotional impact of those elements); (4) cognitive change (changing the cognitions about of the situation to reduce the situation’s emotional impact); and (5) response modulation (directly changing the emotional response). The theory holds that emotion regulation is less effortful and more effective if it occurs at earlier stages of the sequence rather than at the response modulation stage. This model has mainly been applied to intrinsic emotion regulation, and only recently extended to examine extrinsic regulation (e.g., Matthews et al. 2021; Tanna and MacCann 2022).

Extrinsic emotion regulation was defined as “an action performed with the goal of influencing another person’s emotion trajectory; it can aim to decrease or increase either negative emotion or positive emotion” (Nozaki and Mikolajczak 2020, p. 10). We refer to the person who regulates others’ emotions as the *regulator*, and the person being regulated as the *target*. This study considers eight extrinsic regulation processes identified by (MacCann 2022), which were based on Niven et al.’s (2009) classification of interpersonal emotion regulation processes: (1) *downward comparison* (changing the target’s interpretation of the emotion-eliciting event by shifting their frame of reference, specifically by comparing their situation to someone who is worse off); (2) *expressive suppression* (encouraging the target to avoid expressing their feelings in their face, voice, body language or words); (3) *distraction* (focusing the target’s attention away from the situation details triggering their emotions); (4) *humour* (using humour to make the target feel better); (5) *direct action* (changing the target’s situation to alter its emotional impact); (6) *reappraisal* (encouraging the target to change the way they think about their situation in order to change its emotional impact); (7) *receptive listening* (listening to the target express their emotions in socially shared language, typically describing the emotional event that they just experienced or witnessed); and (8) *valuing* (giving the target attention to make them feel valued or special). These processes differ in their levels of engagement with the target’s emotions (i.e., the attention and emotion invested on the target) from low (downward comparison and expressive suppression), to moderate (distraction, humour, direct action), and to high (reappraisal, receptive listening, valuing).

### 1.2. Emotional Intelligence

The major theoretical model of EI is the Mayer–Salovey four-branch model (Mayer et al. 2016) where emotional intelligence comprises four abilities: (1) *emotion perception* (accurately perceiving emotions others’ facial expressions, tone-of-voice, body language, or in evocative creative works); (2) *emotion facilitation of thoughts* (using one’s emotional state to facilitate task performance or goal achievement, which involves both knowing which emotions will be useful for which tasks, and being able to generate those emotions if required); (3) *emotion understanding* (understanding the causes and consequences of one’s own and others’ emotions, and how they are likely to progress over time); and (4) *emotion management* (knowing which actions would be most effective to manage emotions in oneself or others to achieve personal goals). The four branches proceed from the most basic area (emotion perception) to the more cognitive complex area (emotion management) (Mayer et al. 2016; Mayer and Salovey 1997). EI can be assessed with both rating scales (often referred to as ‘trait EI’) and ability scales (often referred to as ‘ability EI’). These are largely separate constructs (despite sharing the same name) and are empirically unrelated (e.g., Joseph and Newman 2010). The current study uses an ability EI framework, and the research below refers to ability EI only.

Ability EI is related to intrinsic emotion regulation processes. Peña-Sarrionandia et al.’s (2015) meta-analysis found that higher EI was associated with lower use of avoidance, rumination, and denial, but higher use of problem solving, reappraisal, and social support seeking. Allen et al. (2015) linked low EI with a decision not to regulate (i.e., not to use any regulation processes) and lower use of situation modification processes but linked high EI with frequent selection of situation modification and cognitive change processes. Megías-Robles et al. (2019) also found that higher EI was associated with lower expressive suppression and higher reappraisal.

However, EI is also expected to affect people’s behaviour in interpersonal contexts (Mayer et al. 2004) and should therefore relate to the processes people use to regulate *other people’s emotions*. When dealing with target’s negative emotions, emotionally intelligent people should be better at identifying what emotions the target has (emotion perception), using the target’s emotions as information to inform their own judgements and behaviours (emotion facilitation), understanding the causes of the target’s emotions (emotion understanding), and managing the target’s emotions (emotion management).

### 1.3. Associations of Emotional Intelligence with Emotion Regulation

Two major axioms drive hypothesis generation in our study. First, because high EI results in greater availability of emotional information about others, it should mean that high EI people are more able and more willing to engage with the emotions of other people. That is, emotionally intelligent people can perceive, understand and use the emotional information to a greater extent. Therefore, EI should show strong positive associations with the high-engagement emotion regulation processes, but negligible or negative associations with the low-engagement emotion regulation processes. Second, because the different branches of EI focus on different abilities, the branch that is most conceptually relevant to regulating emotions (emotion management) should show the strongest associations with extrinsic emotion regulation processes. We outline the logic of these axioms in more detail below.

***EI should relate to highly engaging processes.*** Extrinsic emotion regulation processes differ in the degree to which they require the regulator to devote thought, time, attention and energy to the target’s emotional state and its causes. For example, expressive suppression requires little investment of time or attention—the regulator simply tells the target not to express their emotions. All that is required is that the regulator notices the target is expressing an emotion and evaluates that this is undesirable. In contrast, receptive listening requires active effort on the part of the target. The regulator must pay attention to the target as they express their emotions and make appropriate sympathetic responses, which involves a much greater investment of time and attention. MacCann (2022) classifies the eight extrinsic regulation processes as representing low engagement with the target (downward comparison, expressive suppression), moderate engagement with the target (distraction, humour, direct action) or high engagement with the target (receptive listening, reappraisal, valuing). This classification is supported by correlations of the eight extrinsic regulation processes with empathy and communal orientation—high-engagement processes are positively associated with such variables whereas low-engagement processes show negative or non-significant associations (MacCann 2022; Tanna and MacCann 2022). For example, communal orientation (a tendency to behave in a communal manner by taking others needs into account) showed a significant negative association with downward comparison (*r* = −.32) but significant positive associations with reappraisal, receptive listening and valuing (*r* = .23 to .52).

We propose that people with higher EI have greater capacity to engage with others’ emotions. They can better detect the emotions others feel (emotion perception), know which emotions will be useful for others feel (emotion facilitation), understand what has caused the person to feel that way (emotion understanding) and know which strategies will be effective in regulating their emotions (emotion management). With this knowledge, engaging with others’ emotions is less risky, as emotionally intelligent people are less likely to say the wrong thing and worsen the emotional state. It is also easier, as emotionally intelligent people know what to do. There is evidence that high EI people are more likely to engage with others’ emotions—they have lower attachment avoidance and compassion fatigue but higher empathy (Brackett et al. 2006; Beauvais et al. 2017; Walker et al. 2022). We therefore propose that high EI will show positive associations with the three high-engagement emotion regulation processes (reappraisal, receptive listening, and valuing) and negative associations with the two low-engagement emotion regulation processes (downward comparison and expressive suppression).

***The emotion management branch of EI should show the strongest association with extrinsic emotion regulation processes.*** The emotion management branch of EI is often referred to as *emotion regulation ability* (e.g., Brackett et al. 2010; Extremera and Rey 2015; Ivcevic and Brackett 2014), defined as “the capacity to regulate one’s own and others’ emotional states” (Brackett et al. 2010, p. 407) or the “ability to reason about effectiveness of different emotion regulation strategies” (Ivcevic and Brackett 2014, p. 29). In essence, emotion management is the ability people draw on to decide which emotion regulation processes to select in a given situation. Other branches may influence whether a regulation attempt is made. For instance, you must perceive the target’s emotional state in order to attempt regulation, which involves emotion perception ability. Moreover, you must understand the likely trajectory of the emotion (will it get worse or be long-lasting if nothing is done?) to decide whether a regulation attempt is warranted, which involves emotion understanding ability. However, the *selection of which processes to use* is more clearly related to emotion management than to any other branch of EI. For this reason, we expect extrinsic emotion regulation processes to show stronger associations with emotion management than with the other branches of EI.

### 1.4. Hypotheses

**Hypothesis** **1.** *EI will be significantly associated with extrinsic emotion regulation.*

Specifically, there will be: (a) positive correlations with reappraisal, receptive listening and valuing, and (b) negative correlations with downward comparison and expressive suppression. We have no specific hypotheses about direct action, humor or engagement, but rather examine these associations in an exploratory manner.

**Hypothesis** **2.** *Emotion management will show the strongest prediction of extrinsic emotion regulation.*

When all four EI branches are regressed on each emotion regulation process, the emotion management branch will show the strongest prediction of extrinsic emotion regulation.

## 2. Method

### 2.1. Participants and Procedure

Participants were 179 undergraduate psychology students (35 male, 143 females, 1 nonbinary) recruited from the first author’s institution. As there is only one participant self-identified as ‘nonbinary’, we only included participants who identified themselves as either male or female (*N* = 178) in order to use dichotomous gender as a control variable. Participants were aged between 17 and 47 years (*M* = 19.87 years, *SD* = 3.38 years). Of all the participants, 44% self-identified as Caucasian, 39% as Asian, 10% as Mixed, 4% as Arab, and 3% reported other racial background. They participated in the study for course credit, signed up via the online SONA recruitment system, and completed the assessments online on a device of their choosing. Data analysed in this study and research materials are available at https://osf.io/82r46/?view_only=71c575c8ba4943f3ac715dbd01e8d507.

### 2.2. Measures

#### 2.2.1. Demographic Questions

Participants were asked five demographic questions at the beginning of the survey about their gender, age, proficiency of English, ethnicity, and education level: (1) What gender to you identify as; (2) What is your age in years; (3) How well do you speak English; (4) What ethnicity to you identify with; (5) What is your highest qualification.

#### 2.2.2. Emotional Intelligence

The Mayer-Salovey-Caruso Emotional Intelligence Test (MSCEIT; Mayer 2002) consists of 141 items for 8 subtests (two subtests for each branch of emotion perception, facilitation, understanding, and management). Perception subtests ask participants to rate the extent to which several emotions (e.g., sadness, excitement) are present in: (a) facial expressions (Faces test) and (b) pictures (Pictures test). Facilitation subtests ask participants to rate: (a) how helpful several moods would be for different tasks (Facilitation test) and (b) how similar certain physical sensations are to the feeling of emotions (Sensations test). Understanding subtests ask participants to select which response: (a) represents the best description of complex emotion, based on its constituent emotions (Blends test) or (b) represents the most likely time course for an emotion, given a particular scenario (Changes task). Management subtests present participants with written vignettes of emotional scenarios and ask them to rate how effective several responses would be (for the vignette protagonist) for: (a) regulating their own emotions (Management test) or (b) maintaining a good social relationship with others (Relations test). In this study, we used consensus scoring and considered the total score as well as four branch scores (Emotion Perception, Facilitation, Understanding, and Management). In previous studies of university students, MSCEIT scores related to higher psychological well-being (Brackett and Mayer 2003), life satisfaction (Bastian et al. 2005), empathy (Brackett et al. 2006), and social functioning (Lopes et al. 2005), providing evidence for the validity of the MSCEIT in university student samples.

#### 2.2.3. Extrinsic Emotion Regulation

The Regulating Others’ Emotions Scale (ROES; MacCann 2022) consists of eight four-item subscales, where each item is rated in a 6-point agreement scale ranging from “strongly disagree” (1) to “strongly agree” (6): (1) expressive suppression (I tell them not to frown or cry); (2) downward comparison (“I tell them things could be a lot worse”); (3) distraction (“I divert their attention to something else”); (4) humour (“I do something amusing”); (5) direct action (“I try to fix things for them”); (6) reappraisal (“I help them see events in a new way”); (7) receptive listening (“I listen to them talk about their emotions”); and (8) valuing (“I make them feel special or cared about”). These items are prefaced with the statement “I do the following things to MAKE OTHER PEOPLE FEEL BETTER”. In prior validation studies, the ROES item showed good fit to an eight-factor solution, with scores showed evidence of discriminant validity with respect to broad personality traits, convergent validity with regard to the association with three other emotion regulation assessments, and convergence between self- and informant-reports (MacCann 2022).

## 3. Results

### 3.1. Reliability and Descriptive Statistics

Reliability and descriptive statistics for key study variables are presented in Table 1. Reliability was moderate-to-high for all scales, ranging from .72 (MSCEIT Understanding) to .90 (for Humour). There were significant medium-sized gender differences in two extrinsic emotion regulation processes, with higher scores for females on receptive listening and valuing (*d* = −.53 and −.50, respectively). There were no other significant gender differences. Correlations are presented in Table 2.

### 3.2. Hypothesis 1: EI and Extrinsic Emotion Regulation (Differences among Processes)

Total EI was significantly positively correlated with reappraisal, receptive listening, and valuing whereas significantly negatively correlated with downward comparison and expressive suppression. The effect size was moderate-to-large for receptive listening and expressive suppression, moderate for downward comparison, and small-to-moderate for reappraisal and valuing. However, total emotional intelligence did not have significant correlations with distraction, humour, or direct action. Hypothesis 1 was supported.

### 3.3. Hypothesis 2: EI and Extrinsic Emotion Regulation (Differences among EI Branches)

All the four EI branches were significantly positively correlated with receptive listening and significantly negatively correlated with downward comparison and expressive suppression. Facilitation, understanding and management (but not perception) were significantly positively correlated with reappraisal. Only management (but not the other branches) was significantly positively correlated with valuing. Only understanding (but not the other branches) was significantly negatively correlated with distraction.

Table 3 presents the results of the multiple regressions. Tolerance values ranged from .55 to .67, indicating that collinearity was sufficiently low to allow interpretation of regression coefficients. Only management (and not the other three branches) showed significant prediction of downward comparison, receptive listening, and valuing (supporting Hypothesis 2). Only understanding and management (but not the other branches) predicted expressive suppression (providing some support for Hypothesis 2). For distraction, there was significant positive prediction from the facilitation branch but significant negative prediction from the understanding branch (and no significant prediction from perception or management). Results largely support Hypothesis 2. For the five emotion regulation processes significantly correlated with the total EI score, emotion management was the sole significant predictor for three of them (downward comparison, receptive listening, and valuing), and one of two significant predictors for one of them (expressive suppression). None of the regression coefficients were significant for reappraisal (although the largest effect size was for management).

## 4. Discussion

The present results demonstrated that EI positively correlated with the high emotional engagement extrinsic regulation processes (reappraisal, receptive listening, valuing) and negatively correlated with the two emotion disengagement processes (downward comparison and expressive suppression). Multiple regressions demonstrated that this relationship was largely accounted for by the emotion management branch (except for expressive suppression, where there was also a significant contribution of emotion understanding).

### 4.1. EI Relates to Engagement in Target’s Emotions

Our hypotheses for the EI/extrinsic regulation associations were largely based on the premise that EI would relate to engagement with the target’s emotional state. As we expected, correlations were in different directions for the two processes from the ‘cognitive change’ family—positive associations for reappraisal but negative associations for downward comparison. These two processes are deemed ‘cognitive change’ processes because both change the target’s feelings by changing the way they think. However, downward comparison involves a lower level of engagement in target’s emotions compared to reappraisal, which indicates that less cognitive resources were invested to the target’s emotional information and there is lower willingness to actively regulate the target’s emotions. Moreover, the largest associations were not with the cognitive change family but the response modulation family—positive associations with receptive listening and negative associations with expressive suppression. This suggests that the major link between EI abilities and extrinsic emotion regulation processes is not the *cognitive processes* that produce emotions but the engagement with other people’s *emotions*. Low expressive suppression indicates a willingness to approach or accept people’s negative facial or vocal expressions of emotion whereas high receptive listening indicates a willingness to approach or accept people’s verbal descriptions of their emotions.

Joseph and Newman’s (2010) meta-analysis found that emotion management is the active ingredient of emotional intelligence for predicting job performance, particularly in high emotion labour jobs (those that require ‘service with a smile’). Our results suggest that the pathway for this is through more effective interpersonal interactions. Many jobs involve managing the emotional experiences of other people such as keeping customers satisfied (Kernbach and Schutte 2005), reducing conflict among one’s team members (Jiang et al. 2013), or ensuring that students remain engaged and motivated (Meyer and Turner 2002, 2006). The advantage of high EI may be that workers use more effective processes and fewer ineffective processes for changing others’ emotions—if a core performance indicator is customer happiness, then a person who more frequently uses processes that increase that happiness will be better at their job.

### 4.2. Limitations and Future Directions

Our research used a convenience sample (undergraduate psychology students) in a convenience design (one-off cross-sectional measurement). Future research could expand into different populations, examining the processes used to regulate others’ emotions in romantic relationships, parenting, or high emotion-labour jobs (such as service work, healthcare and teaching) to ensure that findings generalize across different contexts. Our research measured extrinsic emotion regulation by self-rated scales, which reflected only subjective evaluation of the processes that people use to regulate others’ emotions. Future research could adopt an objective approach to measure extrinsic emotion regulation based on behavioural indicators. As extrinsic emotion regulation requires investment of time and attention, emotionally intelligent people may strategically allocate their cognitive and emotional resources. For example, people with high EI do not always use high-engagement processes because they require high levels of cognitive and emotional investment. Therefore, future research could expand the EI/extrinsic emotion regulation associations demonstrated in our research by studying the functional ways through which emotionally intelligent people could effectively regulate others’ emotions. Future research could also examine the relationship between EI and extrinsic emotion regulation using intensive longitudinal methods such as experience sampling or daily diary studies. This is especially important as recent research shows that one-off measurements of ‘trait’ emotion regulation assessments represent not just regulation strategy choice, but also evaluations of the need to regulate and the efficacy of regulation tactics one uses (Koval et al. 2022). It could be that some elements of EI (such as emotion perception) relate to perceiving the need to regulate, some (such as emotion understanding) relate to regulation choice whereas others (such as emotion management) relate to the efficacy of regulation attempts (Double et al. 2022).

### 4.3. Conclusions Remarks

In conclusion, we demonstrate that EI abilities (and particularly emotion management) relate to more effective regulation of other people’s emotions. The strongest links are to the expression rather than suppression of others’ emotions (receptive listening rather than expressive suppression), and to cognitive change processes that focus on positives rather than negatives (reappraisal rather than downward comparison).

## Figures and Tables

**Table 1:** Descriptive statistics, reliabilities, and standard deviations for study variables.

	Alpha	*M*	*SD*
Age	-	19.87	3.38
MSCEIT: Perception	.86	.47	.08
MSCEIT: Facilitation	.77	.42	.07
MSCEIT: Understanding	.72	.49	.07
MSCEIT: Management	.77	.35	.07
MSCEIT: Total	.92	.43	.06
ROES: Downward Comparison	.92	2.68	1.18
ROES: Expressive Suppression	.85	2.34	1.10
ROES: Distraction	.84	4.17	.93
ROES: Humour	.90	4.06	1.17
ROES: Direct Action	.83	4.07	.93
ROES: Reappraisal	.80	4.80	.66
ROES: Receptive Listening	.85	5.42	.63
ROES: Valuing	.86	4.80	.99

Note. MSCEIT = Mayer-Salovey-Caruso Emotional Intelligence Test; ROES = Regulating Others’ Emotions Scale.

**Table 2:** Bi-variate correlations between study variables (N = 178).

	1	2	3	4	5	6	7	8	9	10	11	12	13	14
1. Age	-													
2. Gender (0 = Male; 1 = Female)	−.03	-												
3. MSCEIT: Perception	.09	.04	-											
4. MSCEIT: Facilitation	.08	.19 **	.53 **	-										
5. MSCEIT: Understanding	.10	.02	.46 **	.53 **	-									
6. MSCEIT: Management	.11	.16 *	.38 **	.52 **	.61 **	-								
7. MSCEIT: Total	.12	.13	.76 **	.80 **	.81 **	.79 **	-							
8. Downward Comparison	.06	−.06	−.18 *	−.24 **	−.22 **	−.32 **	−.30 **	-						
9. Expressive Suppression	.06	−.03	−.20 **	−.27 **	−.38 **	−.36 **	−.38 **	.63 **	-					
10. Distraction	−.13	.11	−.04	.05	−.20 **	−.14	−.11	.28 **	.38 **	-				
11. Humour	−.18 *	−.09	.05	.08	−.04	−.05	.01	.23 **	.22 **	.60 **	-			
12. Direct Action	.02	−.12	.08	.02	.07	−.08	.03	.14	.21 **	.32 **	.36 **	-		
13. Reappraisal	−.01	.12	.14	.23 **	.16 *	.23 **	.24 **	−.01	.02	.33 **	.19 *	.36 **	-	
14. Receptive Listening	−.10	.22 **	.21 **	.30 **	.34 **	.43 **	.40 **	−.32 **	−.37 **	.11	.18 *	.13	.30 **	-
15. Valuing	−.01	.22 **	.07	.12	.07	.25 **	.16 *	−.06	−.01	.23 **	.36 **	.22 **	.17 *	.54 **

* *p* < .05, ** *p* < .001.

**Table 3:** Standardized regression coefficients from multiple regressions predicting each extrinsic regulation process from age, gender (0 = male, 1 = female) and all EI branches (N = 178).

	Downward Comparison	Expressive Suppression	Distraction	Humour	Direct Action	Reappraisal	Receptive Listening	Valuing
Age	.10	.11	−.11	−.19 *	.01	−.04	−.15 *	−.03
Gender	.00	.02	.09	−.12	−.10	.06	.15 *	.17 *
Perception	−.03	.00	.00	.05	.08	.01	.02	.01
Facilitation	−.09	−.06	.22 *	.17	.02	.15	.04	−.01
Understanding	.00	−.23 *	−.24 *	−.09	.14	.00	.13	−.10
Management	−.27 **	−.21 *	−.11	−.07	−.19	.14	.31 **	.29 **
*R* ^2^	.12	.19	.10	.07	.04	.08	.24	.10

* *p* < .05, ** *p* < .001.

## Data Availability

The data supporting results reported in this study is available at https://osf.io/82r46/?view_only=71c575c8ba4943f3ac715dbd01e8d507.
